# Directionality in distribution and temporal structure of variability in skill acquisition

**DOI:** 10.3389/fnhum.2013.00225

**Published:** 2013-06-06

**Authors:** Masaki O. Abe, Dagmar Sternad

**Affiliations:** ^1^Research Center for Advanced Science and Technology, The University of TokyoTokyo, Japan; ^2^Department of Biology, Northeastern UniversityBoston, MA, USA; ^3^Department of Electrical and Computer Engineering, Northeastern UniversityBoston, MA, USA; ^4^Department of Physics, Northeastern UniversityBoston, MA, USA; ^5^Center for the Interdisciplinary Research on Complex Systems, Northeastern UniversityBoston, MA, USA

**Keywords:** motor learning, variability, noise, skill, time series analysis, computational model

## Abstract

Observable structure of variability presents a window into the underlying processes of skill acquisition, especially when the task affords a manifold of solutions to the desired task result. This study examined skill acquisition by analyzing variability in both its distributional and temporal structure. Using a virtual throwing task, data distributions were analyzed by the Tolerance, Noise, Covariation-method (TNC); the temporal structure was quantified by autocorrelation and detrended fluctuation analysis (DFA). We tested four hypotheses: (1) Tolerance and Covariation, not Noise, are major factors underlying long-term performance improvement. (2) Trial-to-trial dynamics in execution space exhibits preferred directions. (3) The direction-dependent organization of variability becomes more pronounced with practice. (4) The anisotropy is in directions orthogonal and parallel to the solution manifold. Results from 13 subjects practicing for 6 days revealed that performance improvement correlated with increasing Tolerance and Covariation; Noise remained relatively constant. Temporal fluctuations and their directional modulation were identified by a novel rotation method that was a priori ignorant about orthogonality. Results showed a modulation of time-dependent characteristics that became enhanced with practice. However, this directionality was not coincident with orthogonal and parallel directions of the solution manifold. A state-space model with two sources of noise replicated not only the observed temporal structure but also its deviations from orthogonality. Simulations suggested that practice-induced changes were associated with an increase in the feedback gain and a subtle weighting of the two noise sources. The directionality in the structure of variability depended on the scaling of the coordinates, a result that highlights that analysis of variability sensitively depends on the chosen coordinates.

## Introduction

The past decade has seen a number of studies on motor control and learning that used variability as a window into the underlying processes of skill acquisition. This approach is particularly promising when the task is redundant and affords a manifold of solutions that achieve the desired task result. Such mathematically infinite set of equivalent solutions may be advantageous as the complex sensorimotor system abounds with noise arising at all levels, ranging from variations in ion channel kinetics to amplitudes of action potentials (Faisal et al., 2008). As long as these variations remain within the space of equivalent solutions, the task goal can be achieved.

As early as 1933, Stimpel reported in a throwing task that the release variables showed covariation, such that the throwing precision was better than expected from the individual variables' variability (Stimpel, 1933). More recently, several lines of research have presented support that the sensorimotor system exploits the redundancy of the task by channeling variability into the directions that have no detrimental effect on the task goal (Scholz and Schöner, 1999; Müller and Sternad, 2004, 2009; Todorov, 2004; Cusumano and Cesari, 2006; Cohen and Sternad, 2009; Sternad et al., 2011). For example, using the well-established mathematical concept of null space, the Uncontrolled Manifold (UCM) approach showed that variations in direction parallel to the solution manifold, that are deemed irrelevant to task achievement and, hence, “uncontrolled,” were larger than variability in direction orthogonal to the manifold (Scholz and Schöner, 1999). Hence, the ratio of variances in the two directions expresses the motor system's sensitivity to the solution manifold. A related mathematical approach by Cusumano and Cesari showed similar results (Cusumano and Cesari, 2006). The same concept has been part of the stochastic optimal feedback control framework, where only errors in directions irrelevant for task achievement are penalized by the cost function (Todorov and Jordan, 2002; Todorov, 2004).

Sternad and colleagues developed mathematically different tools in their Tolerance, Noise, Covariation approach (TNC) evaluating variability in terms of its cost to the result, rather than by its covariance in the space spanned by execution variables (Müller and Sternad, 2004, 2009; Cohen and Sternad, 2009). Tolerance evaluates sensitivity to noise in result space, Covariation evaluates the covariation between execution variables, and Noise quantifies the stochastic portion. Parsing the variability into the three components showed that all three contributed to performance improvement, albeit in different degrees: Tolerance improved fastest, while Covariation and Noise had significantly longer time scales (Cohen and Sternad, 2009). Note that unlike the covariance-based approaches, the TNC-analysis differentiates between changes in the overall magnitude of variability or noise and the extent of anisotropy or covariation. It also evaluates changes in the mean, which are outside the scope of covariance-based approaches. The current study complements the TNC-approach by an analysis of directionality in the temporal fluctuations of the data.

Some recent studies added further support to the hypothesis that the CNS channels its excess noise into “do-not-care” directions by examining the temporal structure of data. Projecting individual data in execution space into the directions parallel and orthogonal to the manifold, Dingwell and colleagues showed that the sequential structure in the data showed correlations, i.e., persistence and anti-persistence that differed in the two directions. In their study on treadmill walking, the execution space was defined by stride length and duration with constant (treadmill) speed defining the solution manifold (Dingwell et al., 2010). As hypothesized, the stride-to-stride fluctuations showed anti-persistence orthogonal to the manifold, a finding that was interpreted as error corrections. In a virtual reaching task the same group corroborated the directional differences, but showed persistence in both directions (Dingwell et al., 2012). A recent study on bipedal standing demonstrated higher temporal correlations of postural variability in task-equivalent directions (Verrel et al., 2012). Lastly, van Beers and colleagues reported that in a simple reaching task lag-1 autocorrelations were positive in the task-irrelevant direction, while they were zero in the task-relevant direction (van Beers et al., 2013).

While these studies provided evidence that humans are sensitive to task-relevant directions, several others examined whether this sensitivity is a result of practice. However, surprisingly, the results were not as consistent as expected. For example, Latash (2010) reviews results on UCM-based studies and reports that changes in anisotropy with practice were brought about by a decrease of variability in the orthogonal direction, increase in the parallel direction, or both. Dingwell's temporal analysis of directionality in reaching could not identify changes across two days of practice. As possible causes for these inconsistencies the researchers invoke insufficient duration of practice, or task-related differences, even though task complexity is not a very satisfying explanation. To address the issue of practice duration, the present study will examine performance in the skittles task over 6 days of practice, encompassing familiarization to perfection.

One further possibility for these evident differences in the results may be found in methodological issues that ultimately lead to a conceptual problem. Common to the analyses of data distributions and their fluctuations over time is that the analyses are performed in the space of execution variables. For example, analysis of multi-joint coordination with respect to a single target of the endpoint is analyzed in the space of joint angles; variability in gait speed is analyzed in the space of stride amplitude and duration; throwing accuracy is analyzed in the space spanned by position and velocity at ball release. The underlying assumption is that this space is the space in which the CNS “makes decisions.” This is a daring assumption, as scientists do not yet know the coordinates of the CNS. Sternad and colleagues recently highlighted that the analysis of variability with respect to a null space is highly sensitive to the coordinates that the analysis is conducted in (Sternad et al., 2010). For example, for the UCM-based identification of anisotropy in joint space, the results depend on whether joint angles are defined in relative or absolute coordinates. As it remains unresolved which coordinates the CNS “cares about,” an analysis that depends sensitively on a choice of coordinates may be misguided. Further, if the execution space does not have a metric, orthogonality is not defined. Hence, if directions are not pre-defined, the analysis of directionality is tenuous. This study presents a novel method that identifies the direction of maximal structure of variability in a given space, without an a priori assumption about what is orthogonal to the solution manifold. We will further show by example how rescaling of coordinates can change the results.

In overview, this study will examine skill learning by analysis of variability in both its distributional and temporal structure. We test four hypotheses: (1) Tolerance and Covariation, not Noise, are the major factors underlying long-term performance improvement. (2) Trial-to-trial dynamics in execution space has preferred directions with respect to the solution manifold. (3) This direction-dependent organization becomes more pronounced with practice. (4) The anisotropy in the distributional and temporal structure is in directions orthogonal and parallel to the solution manifold. For the identification of preferred directions in execution space, we will introduce a novel method that is a priori independent of orthogonality.

## Method

### Participants

Thirteen healthy participants (10 males and 3 females, 23–48 years) performed the experimental task after having been given informed consent in accordance with the Institutional Review Board of the Pennsylvania State University. They were right-handed according to the Edinburgh inventory for handedness (Oldfield, 1971). None of the participants had any disorders or injuries in their right limb motor function and they had normal or corrected vision.

### Experimental setup

**Figure 1:**
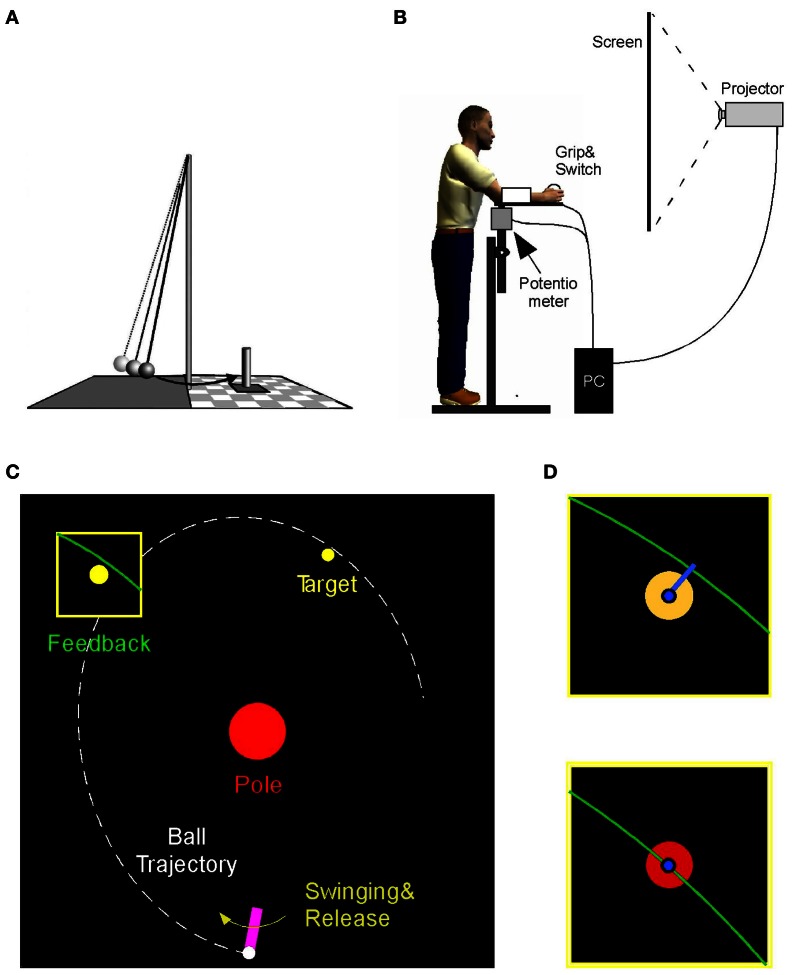
**(A)** Real skittles task. **(B)** The virtual skittle task: participants operated a manipulandum in the horizontal plane that is shown online as a rotating paddle on the visual display. Ball release is triggered by releasing a switch with the index finger. **(C)** Visual display showing the paddle, the ball trajectory, the center post, and the target. The task result was defined as the distance between the trajectory and the target skittle, shown after execution in a zoom window. **(D)** The target color turned red when the distance error was below 1.2 cm.

The experimental task emulated the ball game skittles or tetherball where players throw a ball that is suspended on a string from a vertical post to hit a target skittle on the other side of the post (Figure 1A). The experimental set-up rendered this task in a virtual environment where the participant performed a real forearm movement and initiated the release of a ball by releasing the index finger from a contact switch; the ball only existed virtually (Figure 1B). The ball's trajectory traversed a virtual workspace that was projected for the participant onto a back-projection screen showing a top-down view (Figure 1C). The participant stood ~0.6 m in front of the projection screen (width: 2.50 m, height: 1.80 m). The real-time display showed both the movements of the manipulandum and the ball traversing the center post toward the target. The participant was instructed to hit the center of the target. The error was defined as the shortest distance between the ball trajectory and the center of the target. At the end of a trial the ball's trajectory close to the target was shown in an enlarged window for 1 s after the throw to display the accuracy of the throw (Figures 1C,D). The post in the center of the workspace was represented by a circle of 16 cm diameter. The circular target had a radius of 1.5 cm and was located 50 cm above and 20 cm to the right of the center post. The participant's forearm movement was represented by a solid bar of 12 cm length that was fixed at one end, 50 cm below the center of the post. A circle of 1.5 cm radius representing the ball was “held” and “released” at the free end of the virtual arm by pressing the finger on the contact switch. The display was generated in Visual C++ and projected via an LCD projector (TLP 680U, Toshiba) onto the back-projection screen. The visuomotor delay between the movement and the online display was measured to be 22 ± 0.5 ms.

Participants placed their right forearm on a horizontal manipulandum padded with foam; the participant's forearm was fixed to it with Velcro straps. The height of the manipulandum was adjusted to be comfortable for each participant so that his/her upper arm was at ~45° and the forearm was horizontal. The rotating end of the manipulandum was fixed to a vertical support with its axis of rotation directly underneath the elbow joint. The angular position of the manipulandum was recorded via a 3-turn potentiometer attached to the axis of rotation and was recorded at a sampling frequency of 700 Hz and displayed in real time on the screen (75 Hz update rate).

At the free end of the manipulandum a wooden ball, the size of a tennis ball, was attached. The participant grasped the ball with his/her right hand. A force transducer was attached to the ball located underneath the index finger. To simulate the throw of the ball, the participant moved the arm in an outward horizontal motion and released the ball by extending the index finger, thereby decreasing the force on the sensor. The arm movement resembled that of a Frisbee toss. Both the movements of the arm and the simulated trajectory of the ball were displayed on the screen. The ball's trajectory, as determined by the simulated physics of the task, traversed an elliptic path around the center post as determined by the model equations (see Cohen and Sternad, 2012 for details). This trajectory was not immediately intuitive to participants, and they had to learn the mapping between the real arm movements and the ball's trajectories in the projected workspace. Hence, the task was novel, even for those participants that had experience and skill in throwing.

The ball trajectories were simulated online based on the measured angle and derived velocity at the moment of release. To get the best possible online reading of release velocity and reduce contamination from measurement noise, the last 10 samples of the angular position before the moment of release were fitted with a straight line. This regression slope was used as estimate of the angular velocity at the release moment. This calculation added minimal delay to the display (in the order of 1 μs). To evaluate the error, the minimum distance between the trajectory and the center of the target was calculated.

The elliptic trajectories of the ball were generated by a two-dimensional model in which the ball was attached to two orthogonal massless springs at the origin of the coordinate system (*x* = 0; *y* = 0 in the middle of the post), generating a restoring force proportional to the distance between the ball and the center post. Due to the restoring forces, the ball was accelerated around the center post. At time *t*, the equations for the position of the ball in *x*- and *y*-directions were:

(1) $$x\left(t\right)=A_{x}\sin\left(\omega t+\varphi_{x}\right)e^{-\frac{1}{\tau }}$$

(2) $$y\left(t\right)=A_{y}\sin\left(\omega t+\varphi_{y}\right)e^{-\frac{1}{\tau }}$$

The amplitudes *A*_*x*_ and *A*_*y*_ and the phases φ_*x*_ and φ_*y*_ of the sinusoidal motions of the two springs were calculated from the ball's *x–y* position and velocity at release, which were converted into angle and velocity with respect to the center post. The motions were lightly damped to approximate realistic behavior, with the parameter τ describing the rate of decay for the trajectory (for more detail, see Müller and Sternad, 2004).

### Experimental protocol

For this study participants stood with their shoulder axis at a right angle to the screen, the right shoulder close to the screen. The experimenter instructed participants to throw the ball in clockwise direction performing a forearm rotation as in a Frisbee backhand (see exemplary ball trajectory in Figure 1C). The position of the subject was chosen to make the forearm movement as comfortable as possible to avoid any biomechanical constraints. Aside from the zoomed image of target and trajectory, no explicit quantitative feedback was given. However, if the trajectory passed within 1.2 cm of the center of the target, the target color changed from yellow to red to give a reward signal for successful performance (Figure 1D). The experimenter encouraged participants to achieve as many of these hits as possible. Note that the error distance was always positive, similar to a darts board where the bull's eye is surrounded by iso-error circles.

Participants performed 180 throws per day. After each set of 60 throws, participants were allowed to take a short break. The sequence of throws was sufficiently engaging and the participants reported neither physically nor psychologically fatigued. Each participant performed 180 throws on each of the 6 days. The intervals between collection days were one or two days.

### Analysis of data distributions: tolerance, covariation, and noise

With the goal to quantify how skill changes with practice, the TNC analysis was applied that parses variability into three components. The three components are expressed as costs, quantifying how much of the observed performance error could be improved by a change of Tolerance, Noise, and Covariation (for details see Cohen and Sternad, 2009). Tolerance or T-Cost evaluates how much performance could be improved if the same data distribution were in a more error-tolerant location in execution space. It is calculated by shifting the data in execution space to determine the best location with smallest performance error. Noise or N-Cost is a measure of how random scatter around the mean execution affects performance. It is calculated by shrinking the amplitude of the dispersion toward its mean to determine the scatter that produces minimum error. Covariation or C-Cost quantifies to what degree covariation among execution variables takes advantage of the orientation of the solution manifold. It is calculated by recombining the observed data in execution space and evaluating any improvement in the average results.

### Analysis of directionality in execution space

**Figure 2:**
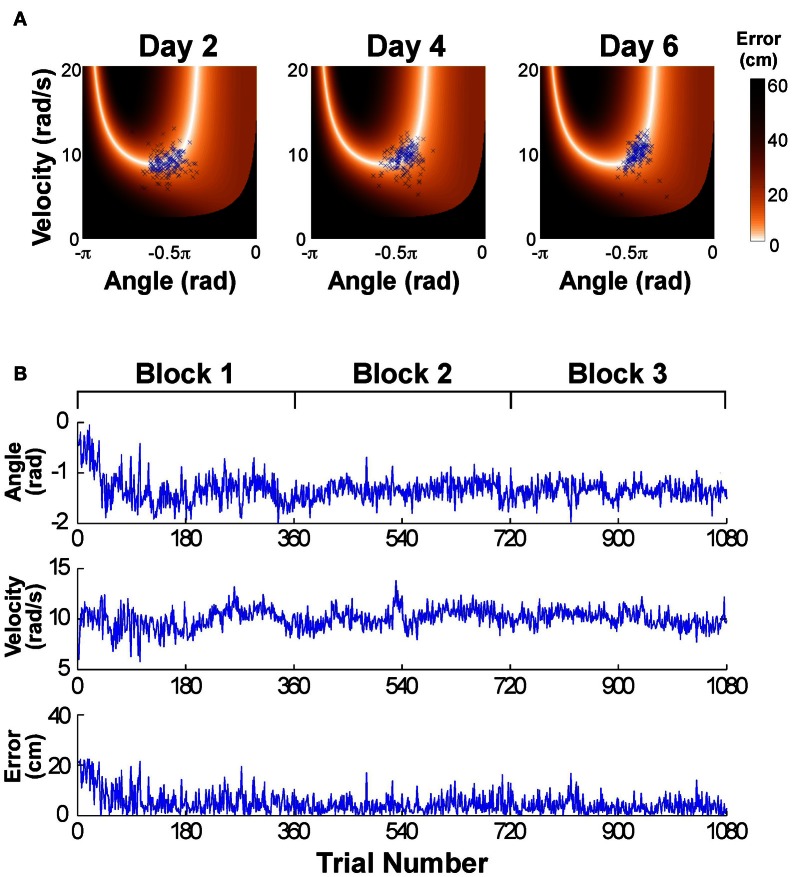
**(A)** Data of one participant represented in execution space. The horizontal and vertical axis shows position and velocity, respectively, and the color encodes the error. The data distribution changes across days by decreasing the amplitude of scatter and its distribution/covariation with respect to the solution manifold. **(B)** Typical time series of angular position, velocity, and error across the 6 experimental days.

Figure 2A shows the data distributions of 3 days plotted in execution space spanned by angular position and velocity of the paddle; the color shades code the magnitude of error for all position-velocity combinations if the ball were released at this position-velocity combination. The set of zero-error solutions defines the solution manifold, which is a one-dimensional set shown in white. The black areas indicate position-velocity combinations, i.e., ball releases, where the ball would hit the center post. The blue symbols are the 180 throws per day showing a small decrease in scatter with practice, concentrating increasingly more on the light-colored area, where errors are small or 0; on day 6 a more elliptic distribution in alignment with the solution manifold is visible. For the time series analysis the data of the six practice days were first pooled into three blocks to ensure a sufficient number of data: Block 1: day 1 and 2, Block 2: day 3 and 4, Block 3: day 5 and 6. Figure 2B shows the associated time series of the execution variables (position and velocity) and the result variable (error) across the entire 6 days (or three blocks) with 1080 throws.

The time series analyses on the directionality of changes from trial to trial were conducted in execution space spanned by angular position and velocity. However, due to the different units of position and velocity, distance, and orthogonality are not defined in this space (Sternad et al., 2010). A commonly used procedure to overcome this problem is to normalize the units by dividing the variables by their standard deviations:

(3) $$x_{1}\left(i\right)=(p\left(i\right)-\bar{p})/\sigma_{p}$$

(4) $$x_{2}\left(i\right)=(v\left(i\right)-\bar{v})/\sigma_{v}$$

where *x*_1_(*i*) and *x*_2_(*i*) denote normalized position and velocity, *i* is the trial number, *p*(*i*) and *v*(*i*) position and velocity, $\bar{p}$ and $\bar{v}$ are means of one block, and σ_*p*_ and σ_*v*_ are standard deviations of position and velocity of the same block. Note that this procedure assumes that covariance can be used to define a metric and that the metric only has diagonal entries. This normalization was performed for each participant and each block separately.

**Figure 3:**
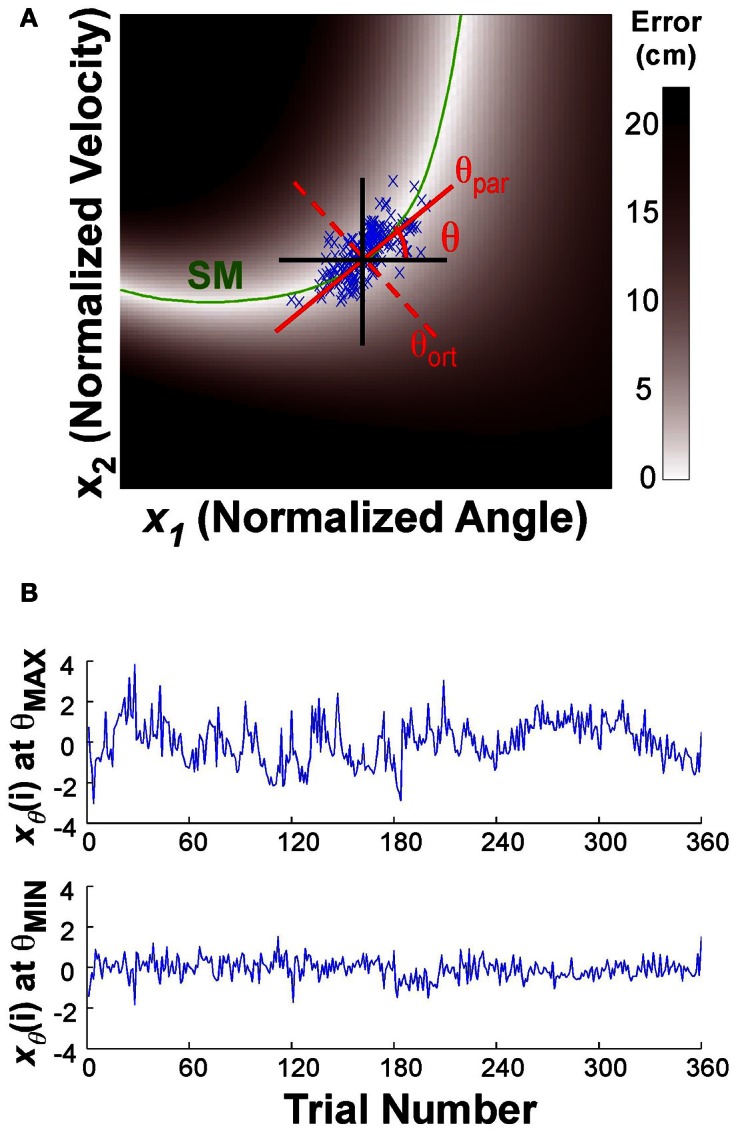
**(A)** Execution space and illustration of the rotation axis used to analyze temporal fluctuations in different directions. The execution space was normalized by the individual subject's standard deviations. The green line shows the solution manifold (SM); the black lines show the directions parallel to the x- and y-axes; the red lines show the rotation axes parallel to and orthogonal to the solution manifold, respectively. The one-dimensional time series *x*_θ_(*i*) is obtained by projecting the variables onto the rotation axis as expressed in the equation. **(B)** The time series at the top shows *x*_θ_(*i*) for the rotation angle θ with the maximum autocorrelation value, the bottom time series shows *x*_θ_(*i*) for the rotation angle θ with the smallest autocorrelation.

To assess whether the trial-to-trial changes had a directional preference, the data of one block were projected onto a line through the center of the data set:

(5) $$x_{\theta }\left(i\right)=x_{1}\left(i\right)cos\theta +x_{2}\left(i\right)sin\theta$$

where *x*_θ_(*i*) denotes the new variables after projection onto the line. The angle θ of this line was defined as 0 when parallel to the x-axis or position direction; θ = 0.5π rad when parallel to the y-axis or velocity direction (Figure 3A). The direction parallel to the solution manifold was defined as θ_par_ for each individual; the direction orthogonal to the solution manifold was defined as θ_ort_. The center of the data was defined by the median of the position and the median of the velocity data for each block of each individual (the median was chosen to avoid any bias from outliers). This line was then rotated through 0 < θ < π rad, in 100 steps of 0.01^*^π rad. At each rotation angle, the data were projected onto the line (Equation 5) and the time series of the projected data was evaluated as described next. Note that this point of rotation was close, but not exactly on the solution manifold, especially early in practice. (The average distance from the solution manifold measured in terms of error was 1.21 ± 1.0 cm.)

### Analyses of time series

We evaluated the temporal structure of *x*_θ_(*i*) for each rotation angle θ of the line through the center of the data set. At each angle both autocorrelation and detrended fluctuation analysis (DFA) were computed. From the autocorrelation analysis, only the lag-1 coefficient (abbreviated as AC1) was reported. To assess temporal structure beyond lag-1 the DFA was evaluated. DFA is a modification of the root-mean square analysis of a random walk (Feder, 1988) but is less sensitive to non-stationarities and noise in the data.

For the DFA analysis, the time series was first cumulatively summed to obtain an integrated time series; this integrated series was linearly detrended within a given window *n*. The root mean square value of the detrended time series *F*(*n*) was calculated for different window sizes *n*. Plotting *F*(*n*) over *n* on a log-log scale the scaling index SCI was obtained from the slope of the linear regression of log*F*(*n*) over log(*n*). This scaling index quantifies the long-range correlations of the time series. If SCI = 0.5, the time series has no time correlation as in white noise. If 0.5 < SCI < 1.0, the time series is categorized as a stationary signal with fractal noise (Eke et al., 2002). In this case, he increasing and decreasing tendency of the time series persists. Using sets of 60 trials the slope was calculated for window sizes between 6 and 20 trials. Although this size of the samples is relatively short compared to other applications of the DFA analysis, we opted for this size to avoid discontinuities that may arise from subjects taking short breaks. We calculated both AC1 and SCI in the time series of angular position, velocity, and error. Figure 3B shows two time series of the projected data for the two directions θ that showed the minimum and maximum values of the autocorrelation analysis (which was very close to the minimum and maximum of the DFA). The difference in fluctuation profile is visible by eye. For comparison, autocorrelation and DFA analyses were also performed on surrogate data. These surrogate data were produced by randomly shuffling the time series. These analyses were conducted 20 times to obtain mean results and standard deviations similar to the data.

### Statistical analyses

The changes in error and T-Cost, C-Cost, and N-Cost across practice were fitted by exponential functions to assess the different time scales of change. Pearson correlations between TNC-Costs and error revealed contributions of the costs to error. The directionality analysis of AC1 and SCI, specifically its maximum and minimum values, θ_max_ and θ_min_, were analyzed with *t*-tests and Smirnov-Grubbs tests to compare them against directions of the solution manifold and the angle and velocity direction. Two-way repeated-measures ANOVAs were used to assess changes with practice. The significance level was set to 0.05. Analyses were conducted with SPSS v16.

## Results

### Performance improvement

**Figure 4:**
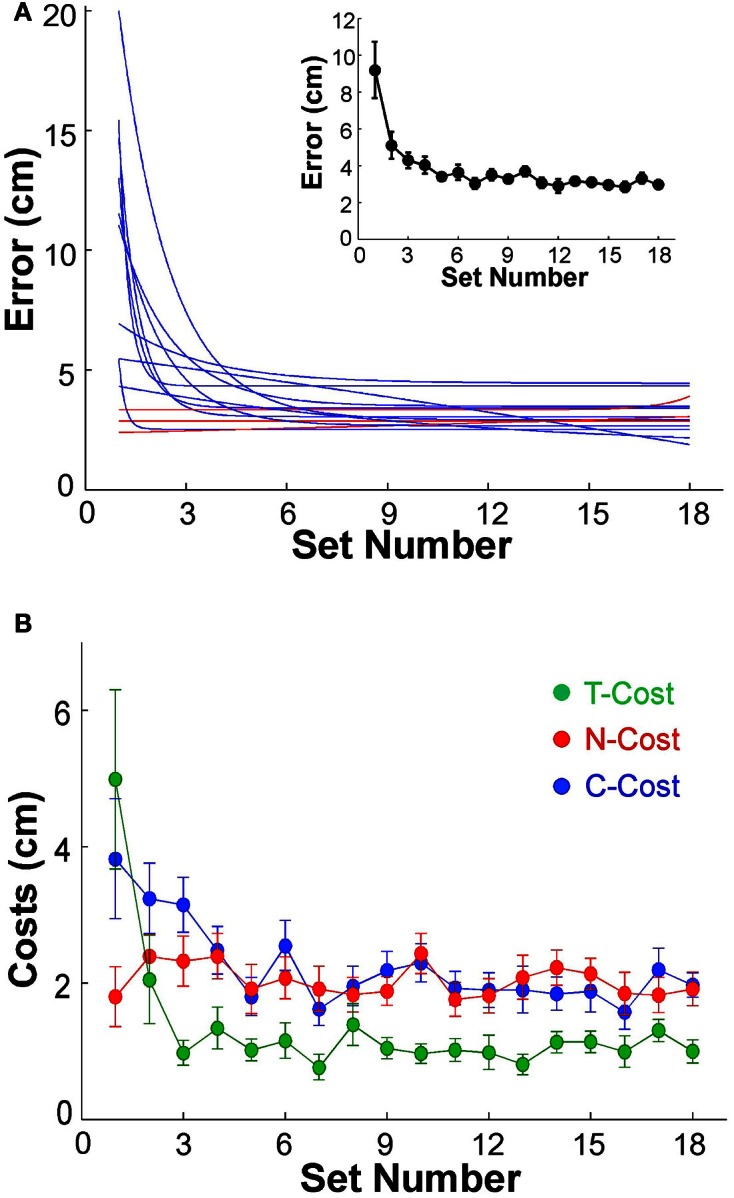
**(A)** Performance errors for all 13 participants across 18 sets of 60 trials each. The blue and red lines represent the exponential fits for all 13 individuals. Red lines indicate the three subjects that did not show any improvement across practice. The insert shows the average error across all 13 subjects; the error bars denote standard errors across participants. **(B)** Participant averages of T-Cost, C-Cost, and N-Cost across all 18 sets in the 6 days of experiments.

Before analyzing variability as a function of practice we first assessed whether participants indeed showed the expected performance improvement. The average error, or distance to the target, in sets of 60 trials was plotted to establish that participants showed the expected learning. The individual error profiles of the 13 subjects were fitted by exponential functions and are summarized in Figure 4A (to avoid clutter, data points are not shown). While 3 participants performed with small error from the beginning of practice and showed no improvement (P2, P6, P12, shown by red lines), 10 individuals showed a visible decrease in error; the *R*^2^-values of their exponential fits were between 0.26 and 0.95. The inset of Figure 4A shows the average decrease of error of all 13 participants across the 18 sets. From an initial 9.19 cm in the first set, the average error declined exponentially to 2.98 cm in the final set; the *R*^2^ of the exponential fit was 0.96, the time constant was 1.07. Subsequent analyses were conducted with both inclusion and exclusion of the three individuals who showed no improvement, but the statistical results were not affected.

### Tolerance, covariation, and noise

Figure 2A showed an exemplary participant's data distributions in execution space on Days 2, 4, and 6. As could be seen, the relatively isotropic data distributions on Day 2 and Day 4 showed a visible change on Day 6, where the data started to cluster along the solution manifold. Interestingly, the data showed little decrease in the overall amplitude of dispersion. This observation was quantified by T-Cost, C-Cost, and N-Cost. Figure 4B shows the three costs averaged over all participants across the 18 practice sets. T-Cost shows a rapid decline and reaches a plateau at set 3, which corresponds to the end of day 1. C-Cost also shows a visible decline which lasts over the first 6 sets, leading to a plateau thereafter. N-Cost did not show any obvious improvement. To directly test whether error was reduced via changing C-Cost, Pearson correlations were performed between error and T-Cost, C-Cost, and N-Cost for each participant. Table 1 summarizes these results: as expected, both T-Cost and C-Cost showed significant positive correlations with error in most participants. While causal conclusions cannot be made, these results nevertheless strongly suggest that Tolerance and Covariation in the execution variables lead to the observed reduction in error. In contrast, N-Cost only showed 4 significant positive correlations. Three of these correlations were seen in the 3 participants that showed low error scores right from the beginning (P2, P6, P12). This suggests that small changes in N-Cost may still account for some of the changes across the trial sets in the three very good subjects. In sum, these results were consistent with Hypothesis 1.

**Table 1:** **Results of correlations between error and T-Cost, C-Cost, and N-Cost (Pearson correlation coefficients *r*)**.

**Participant**	**T-Cost**		**C-Cost**		**N-Cost**	
	***r***	**Sig**	***r***	**Sig**	***r***	**Sig**
1	0.979	^***^	0.923	^***^	0.368	
2	0.734	^***^	0.773	^***^	0.808	^***^
3	0.774	^***^	0.778	^***^	0.148	
4	0.914	^***^	0.981	^***^	−0.041	
5	0.961	^***^	0.177		−0.157	
6	0.639	^**^	0.481	^*^	0.524	^*^
7	0.966	^***^	0.205		−0.138	
8	0.915	^***^	0.747	^***^	−0.118	
9	0.729	^***^	0.479	^*^	0.267	
10	0.591	^**^	−0.061		0.248	
11	0.934	^***^	0.930	^***^	0.370	
12	0.831	^***^	0.313		0.630	^**^
13	0.904	^***^	0.591	^*^	0.565	^*^
Mean	0.836		0.563		0.267	
SD	0.130		0.330		0.316	

Participants 2, 6, and 12 are the ones that did not show any improvements in the error measure (Figure 4).

* p < 0.05;

** p < 0.01;

*** p < 0.001.

### Autocorrelation and scaling index

**Figure 5:**
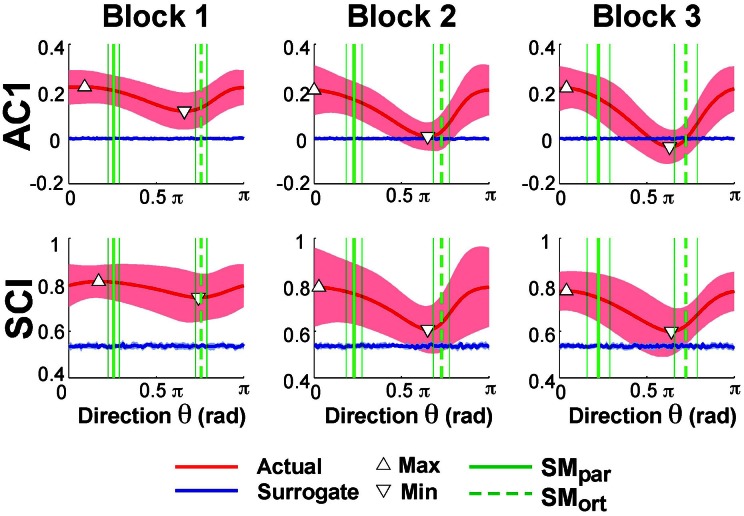
**Lag-1 autocorrelations AC1 of *x(i)* and scaling index SCI of the detrended fluctuation analysis as a function of rotation angle across the three blocks**. The solid red lines show the average modulation of AC1 and SCI across all participants and the shaded areas represent one standard deviation around this mean. The triangles denote the minimum and maximum value of AC1 and SCI; the vertical green lines are the direction of the solution manifold with its variations across participants, denoted by the dashed lines. The horizontal blue lines are the results of the surrogate analyses.

Exemplary time series at the minimum and maximum value of AC1 were already presented in Figure 3B to visualize that the structure of their fluctuations was different. Figure 5 summarizes the results of AC1 and SCI as a function of direction θ. Note that θ = 0 rad was defined as parallel with the x- or position-axis and θ = π/2 rad was parallel with the y-axis or velocity in execution space. Hence, the orthogonal and parallel direction, indicated by the green vertical lines, differed for each subject as they centered their data at slightly different locations with respect to the solution manifold. The six panels show the average AC1 and SCI over all participants for each direction θ across the three blocks; the shaded areas around the solid red line indicate one standard deviation across all participants.

While AC1 was predominantly positive, the values also showed a clear modulation with θ, especially in Blocks 2 and 3. Similarly, SCI was consistently between 0.5 and 1.0 and showed an equivalent modulation with θ. The directions at which AC1 and SCI reached their minima and maxima, θ_min_ and θ_max_, are indicated by triangles. The blue lines show the results for the time-shuffled surrogate data with each value representing an average from 20 repeated shuffles. As expected, these results did not show any modulation across θ and were close to 0 and 0.5, respectively. Hence, the data showed persistence in all directions but of varying degree, as stated in Hypothesis 2.

**Figure 6:**
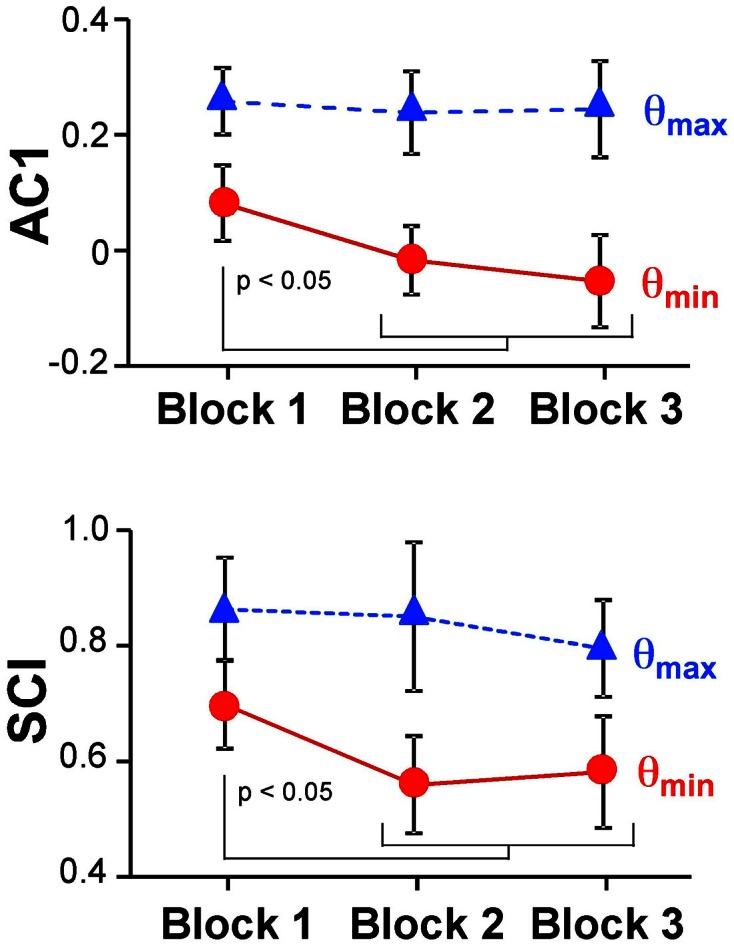
**Average results of the autocorrelation AC1 and scaling index SCI at maxima and minima directions across the three blocks**.

Figure 6 summarizes the changes of the AC1 and SCI minima and maxima across the three blocks showing the means across participants and their standard deviations. Both extrema of AC1 at θ_max_ and θ_min_ were subjected to a 3 (block) × 2 (variable) repeated-measures ANOVA. It rendered a significant interaction, *F*_(2, 24)_ = 4.69, *p* = 0.019, and both main effects were significant: block, *F*_(2, 24)_ = 7.43, *p* = 0.003, and variable, *F*_(1,12)_ = 264.96, *p* < 0.001. *Post-hoc* tests showed that AC1 at θ_min_ decreased significantly from Block 1 to Block 2 and to Block 3 (*p* < 0.05). These observations were consistent with Hypothesis 3. In contrast, AC1 at θ_max_ did not show significant changes across blocks. The same ANOVA for SCI showed equivalent results: the interaction was significant, *F*_(2, 24)_ = 7.85, *p* = 0.002, as were the main effects for block, *F*_(2, 24)_ = 8.89, *p* = 0.001, and variable, *F*_(1, 12)_ = 202.23, *p* < 0.001. The values of θ_min_ changed significantly from Block 1 to Block 2 and to Block 3 (*p* < 0.05), while θ_max_ did not show any significant differences among blocks.

In Hypothesis 4 we stated that long-range correlations should be maximal in the direction parallel to the solution manifold and minimal in the direction orthogonal to the solution manifold. Returning to Figure 5 shows *SM*_par_ and *SM*_ort_ averaged across all participants depicted by the green vertical lines; the thin lines indicate one standard deviation across all participants. To test Hypothesis 4 the angles of *SM*_par_ and θ_min_ and of *SM*_par_ and θ_max_ were computed for each subject and compared by pairwise *t*-tests. The results were only partially consistent with this hypothesis: the minima were close to *SM*_ort_, while the maxima significantly differed from *SM*_par_. The average angle differences between *SM*_ort_ and θ_min_ across all subjects and all three blocks were: 0.24 ± 0.39 rad for AC1 and 0.23 ± 0.42 rad for SCI. The average differences between *SM*_par_ and θ_max_ across all three blocks were 0.55 ± 0.45 rad for AC1 and 0.36 ± 0.65 rad for SCI. These differences were statistically significant from zero (*p* < 0.01) and did not show any changes across blocks. These results were not consistent with Hypothesis 4.

To further assess whether the observed extrema indicated sensitivity to the solution manifold as hypothesized, or whether they were merely coincident with the measured variables angular position and velocity, Smirnov-Grubbs tests evaluated whether θ_min_ and θ_max_ differed from the position or velocity direction, 0 or π/2 rad, respectively. Results showed that for both AC1 and for SCI θ_min_ was not significantly different from π/2 rad (velocity) in Block 1, but differed in Block 2 and Block 3 (*p* < 0.01). The autocorrelations at θ_max_ were not significantly different from AC1 in the position direction or 0 rad in all blocks (*p* > 0.05). In sum, the direction of maximum persistence was observed in the angle direction.

## Modeling

The observed results showed significant changes in the structure of variability, both in distributions and in their temporal fluctuations. However, several aspects in the time series analyses also deviated from the expectations formulated in Hypotheses 2 and 4: the autocorrelations were overall positive (counter Hypothesis 2), and the maxima and minima in the temporal structure deviated from the parallel and orthogonal directions defined by the solution manifold (counter Hypothesis 4). A simplified model of skittles was used to shed light on these unexpected observations and suggest possible mechanisms that account for them. The model will not only reproduce the positive autocorrelations and its changes with practice, it will also show how a rescaling of the execution coordinates may sensitively skew the results, highlighting that reliance of the analysis on a pre-defined orthogonality in execution space may be misguided.

**Figure 7:**
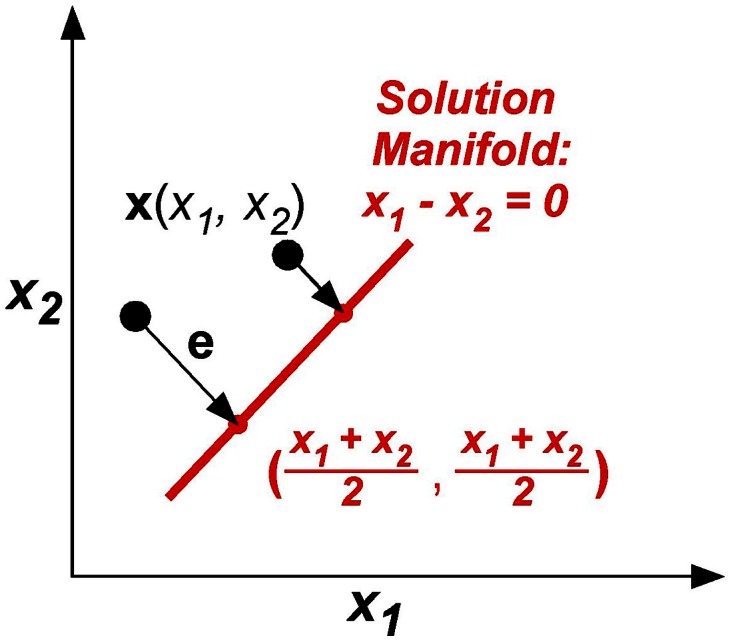
**The model task: two execution variables (*x*_1_, *x*_2_) and one result variable e are defined in execution space**. Error **e** is defined by minimum distance between **x** and **x**_Target_ in execution space.

The model was kept as simple as possible, yet captured the essential component of the skittles task—redundancy. The simplification made the task similar to a line-reaching task: There were two execution variables *x*_1_ and *x*_2_ (like position of an endpoint in the plane) and there was one result variable, the error *e*, or distance from reaching the line (Figure 7). The task was to change execution (*x*_1_, *x*_2_) to be on the line, defined by *x*_1_ – *x*_2_ = 0. The error *e* or result variable was defined as the minimum distance between the execution (*x*_1_, *x*_2_) to the solution manifold. Note that this error definition simplifies the skittles task as it excludes the dynamics of the ball trajectory. In skittles, the ball dynamics creates an approximately parabolic increase of the error orthogonal to the solution manifold; the linearization in this simplified model is acceptable for a sufficiently small neighborhood. Importantly, the model has redundancy, mapping two execution variables into one result variable, analogous to the skittles task.

To simuate trial-by-trial learning and the increasing anisotropy in the data distributions with practice, the main assumption was that the execution variables **x**(*i*) = [*x*_1_, *x*_2_]^*T*^ were updated based on the previous states. The error was defined as **e**(*i*) = **x**(*i*) – **x**_Target_, where **x**(*i*) was the output state and **x**_Target_ was the target state defined in the workspace. The target state defined the point closest to **x**(*i*) on the line or solution manifold:

(6) $$x_{\text{Target}}={\left[\frac{x_{1}+x_{2}}{2},\frac{x_{1}+x_{2}}{2}\right]}^{T}$$

Consequently, the error was defined as:

(7) $$e\left(i\right)=x\text{​}\left(i\right)-x_{\text{Target}}={\left[\frac{x_{1}-x_{2}}{2},\frac{x_{2}-x_{1}}{2}\right]}^{T}$$

The motor command **u**(*i* + 1) was obtained by subtracting the error signal *e*(*i*) from the previous *i*th motor command **u**(*i*). The error was weighted by the feedback gain ***B***. Two sources of additive noise were included: *r*_*E*_ was added to the motor command **u**(*i*) at the execution level; at the planning level *r*_*P*_ was added to obtain the updated command **u**(*i* + 1) (van Beers, 2009). Both noise sources were independently drawn from a Gaussian distribution with 0 mean and unit amplitude η(*i*). The coefficient ω defined the relative magnitude of the two noise sources *r*_*P*_ and *r*_*E*_. The model is summarized as follows:

(8) $$\begin{array}{l} \;x\text{​}\left(i\right)=u\text{​}\left(i\right)+r_{E}(i)\\ \;e\left(i\right)=x\text{​}\left(i\right)-x_{\text{Target}}\\ u\text{​}\left(i+1\right)=u\text{​}\left(i\right)-Be\text{​}\left(i\right)+r_{P}(i+1)\\ \;r_{P}\text{​}\left(i\right)=\omega \eta_{1}(i)\\ \;r_{E}\text{​}\left(i\right)=(1-\omega )\eta_{2}(i) \end{array}$$

In forward simulations, 50 values for the feedback gain ***B*** (between 0 and 0.5), and 20 values for the relative noise magnitude ω (between 0 and 1) were tested. For each of the 1000 parameter combinations we simulated 100 runs with different initial values for the noise sources *r*_*E*_ and *r*_*P*_; the initial value for **u**(0) was always (0, 0). For each simulation output, autocorrelations AC1 were calculated for all direction angles, using the same procedure as for the experimental data. Given that the autocorrelation analysis and the DFA rendered consistent results in the experimental data, the analyses were confined to the autocorrelations.

## Simulation results

**Figure 8:**
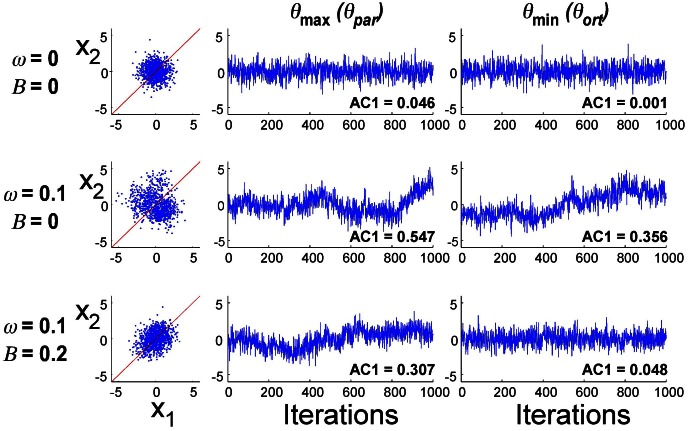
**Exemplary simulation results for three different parameter combinations (1000 iterations)**. Distribution of data in execution space and time series of error in two directions in execution space.

Exemplary data distributions and time series in the principal directions for three different parameter combinations are presented in Figure 8. The first simulation result with *B* = 0 and ω = 0 illustrates the case where planning noise *r*_*P*_ was 0 and there was no error fed back to the update of **u**(*i*). Not surprisingly, the distribution in *x*_1_–*x*_2_-execution space was isotropic and the time profiles over 1000 iterations of the error signal in both parallel and orthogonal directions were random, as indicated by the AC1 values close to 0. The second row illustrates how the presence of the second noise source changed the distribution and the temporal structure of the noise: the distribution became larger and the autocorrelations in the two directions became positive. Note that the feedback gain *B* was still 0. The third row illustrates the case where both noise sources (ω = 0.10) and error feedback (*B* = 0.20) were present: the distribution shows covariation and the autocorrelations parallel to the solution manifold were positive, while they were close to 0 in the orthogonal direction. Despite these significant differences in distribution and temporal structure in the three parameterizations, the overall magnitude of the variability was similar.

**Figure 9:**
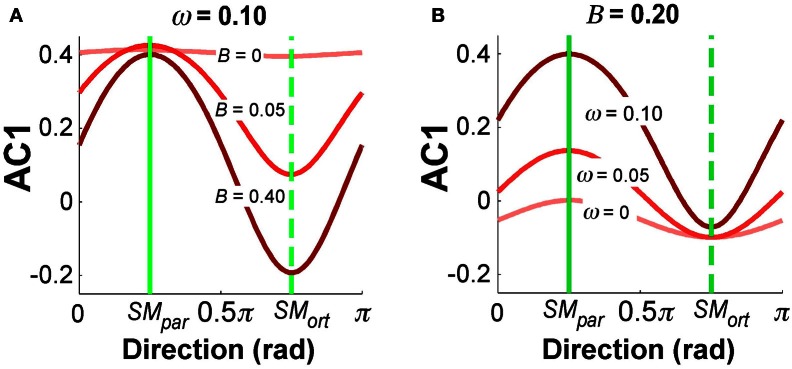
**Simulation results for autocorrelation AC1 as a function of direction angle. (A)** *B* = 0.20 and ω = 0, 0.05, 1.0. **(B)** ω = 0.1 and *B* = 0, 0.05, 0.40. The values depict average results from 100 simulation runs for each direction angle.

Figure 9 summarizes the simulation results for selected parameter combinations in the same format as the data summary in Figure 5. Setting ω = 0.10 as in Figure 8, Figure 9A illustrates the values of AC1 across all direction angles θ for three different feedback gains *B*. The parallel and orthogonal directions with respect to the solution manifold were symmetric at 0.25π and 0.75π rad. The simulations revealed that the magnitude of *B* selectively affected AC1 in *SM*_ort_: zero feedback gain led to positive AC1 values in directions *SM*_par_ and *SM*_ort_; for increasing feedback gains the modulation of AC1 at *SM*_ort_ became more pronounced and AC1 became slightly negative. This is intuitive and reflects the increasing influence of corrections that minimize the error. Figure 9B depicts the effect of the relative noise amplitudes ω on AC1, keeping *B* fixed at 0.20. The modulation of AC1 at *SM*_par_ showed a decrease of AC1 for smaller ω. Hence, the experimentally observed modulations across direction angles and the signs of AC1 reflect the relative magnitude of the noise and feedback parameters.

**Figure 10:**
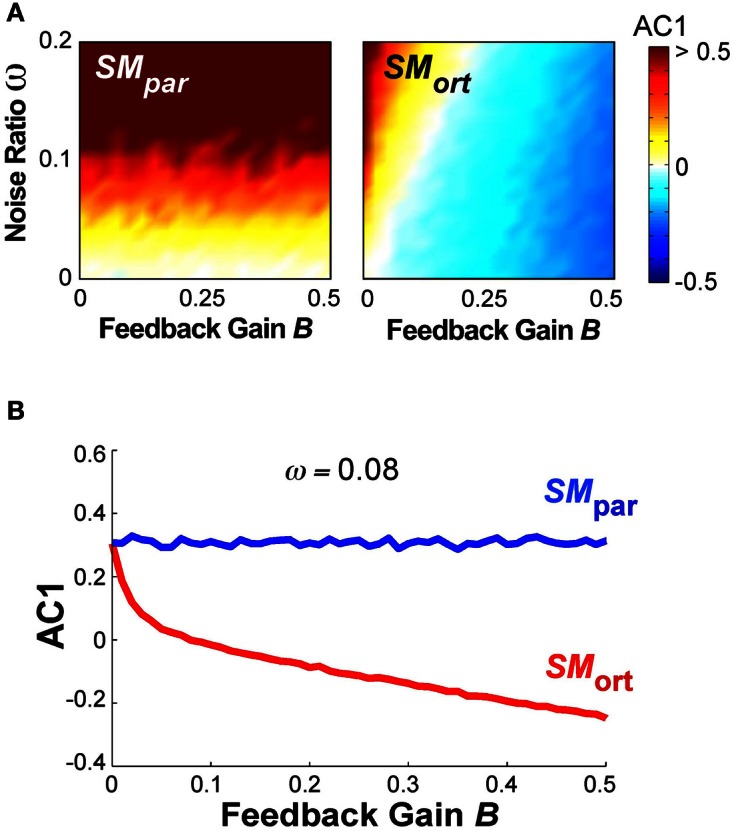
**(A)** Simulation results for lag-1 autocorrelation AC1 at θ_par_ and θ_ort_ as a function of feedback gain *B* and relative noise ratio ω. Color bar (right) represents the autocorrelation values. **(B)** Simulation result for lag-1 autocorrelation (AC1) at θ_par_ and θ_ort_ as a function of *B* with ω = 0.08 that shows a similar pattern as the experimental results as a function of practice.

A different summary of the AC1 results for all *B* and ω parameter combinations is shown in Figure 10A, results at *SM*_par_ are shown in the left panel, at *SM*_ort_ in the right panel. The magnitude of AC1 is represented by color, with red showing positive values and blue showing negative values. At *SM*_par_ AC1 was mainly affected by the noise ratio ω; at *SM*_ort_ AC1 was affected by both variables ω and *B*. As is to be expected, the larger the feedback gain *B*, the more negative the autocorrelations in the orthogonal direction. For small feedback gains, the noise ratio has a significant effect on AC1, which disappears at higher values of *B*.

Figure 10B shows the magnitude of AC1 at *SM*_par_ and *SM*_ort_ for a constant ω = 0.08. The specific ω-value was chosen because it generated similar AC1 results as seen in the experimental data. When AC1 was plotted as a function of feedback *B*, the figure shows that AC1 at *SM*_ort_ decreased, while AC1 at *SM*_par_ maintained almost the same value throughout. This pattern was qualitatively and quantitatively similar to the change of AC1 at θ_max_ and θ_min_ in the experimental results (Figure 6). It suggests that changes in performance were mainly brought about by changes in the feedback gain.

One important observation is that, different from the experimental results, the minima and maxima of AC1 in Figure 9 were exactly at 0.25π and 0.75π rad. This is to be expected for the linear manifold that is defined at 0.25π rad (45°) in execution space. Furthermore, the simple model assumed an execution space with two variables of the same units such that the space had a metric and orthogonality was defined. This contrasts with the experimental case where the two execution variables had different units and normalization was applied to allow for a distance measure and definition of angle. However, this normalization is necessarily a crutch as we do not know the true metric of the variables inside the nervous system.

To illustrate how a scaling of the variables may thwart orthogonality and thereby the minima and maxima of the temporal structure, we performed model simulations with different types of rescaling of the execution variables. To emulate the case where the state variables may be rescaled “inside the CNS”, we conducted simulations where **x**(t) was rescaled at each iteration. Specifically, we included a rescaling of *x*_1_: *x*′_1_(*i*) = α (*u*_1_(*i*) + *r*_*E*_), where α is the scaling factor. Setting the system parameters to *B* = 0.10 and ω = 0.08, we performed the simulations with α = 2 and α = 0.5. In a first set of simulations the solution manifold was not changed. This case emulated the interpretation that the solution manifold was defined in external physical space, where the units are given. In a second set of simulations, the solution manifold was adapted to the rescaling of variables.

**Figure 11:**
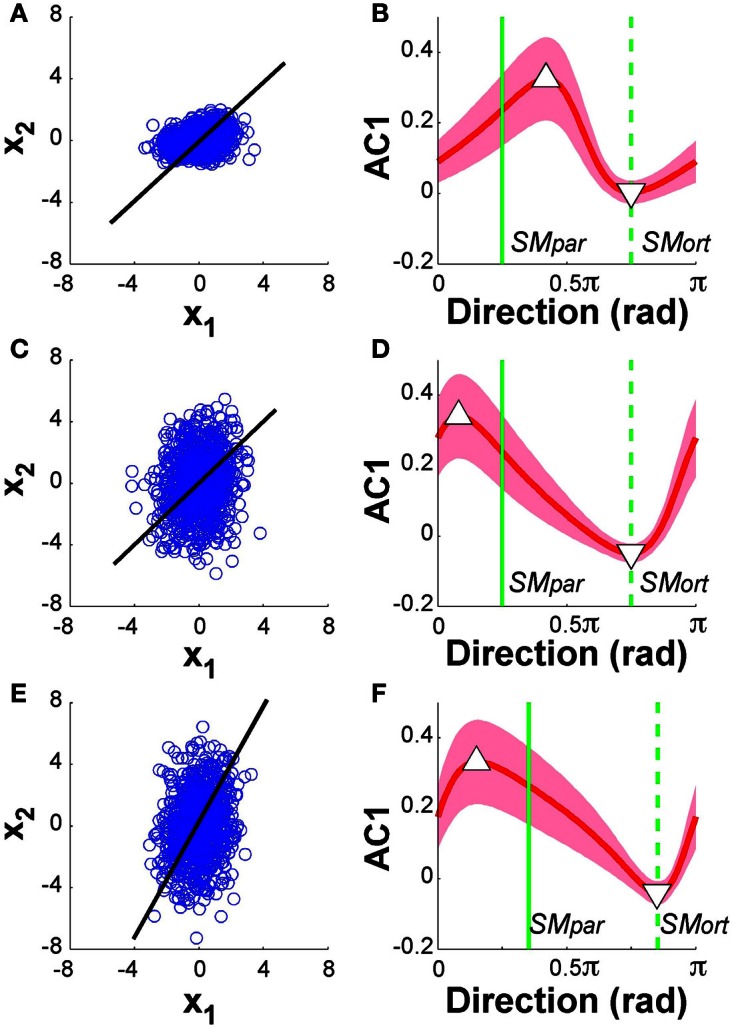
**Model analysis with one of the two execution variables scaled**. The left panels show the data distributions of 1000 simulation runs plotted in execution space. The black line denotes the solution manifold. The right panels summarize the results of the autocorrelation analysis plotted as a function of direction. The red lines with the shaded bands represent the mean and standard deviations across 1000 simulations for each of the 100 directions; the green vertical lines denote the parallel and orthogonal direction of the solution manifold. Panels **(A)** and **(B)** show the simulation results with SM: *x*_1_ = *x*_2_, α = 0.5; panels **(C)** and **(D)** shows results with SM: *x*_1_ = *x*_2_, α = 2; panels **(E)** and **(F)** show the case of SM: *x*_1_ = 2*x*_2_ and α = 2. In all cases, the minima and maxima of the autocorrelations, marked by triangles, have shifted away from the orthogonal and parallel directions of the solution manifold. The simulations in panel **(D)** show very similar results to the experimental data in Figure 5.

Figure 11 summarizes the results: the panels on the left show the data from 1000 runs in execution space together with the linear solution manifold (black line). The panels on the right display AC1 of the time series as a function of direction, in the same format as the experimental and model data in Figures 5, 9, respectively. The red line represents the mean of 1000 runs for each of the 100 directions, the green vertical lines denote the parallel and orthogonal directions of the solution manifold. Panels (A) and (B) show the simulation results with SM: *x*_1_ = *x*_2_, α = 0.5; panels (C) and (D) shows results with SM: *x*_1_ = *x*_2_, α = 2; panels (E) and (F) show the case of SM: *x*_1_ = 2*x*_2_ and α = 2. Note that in Figure 11D, the parallel and orthogonal directions of the solution manifold were unchanged, while they were shifted in Figure 11F. The minima and maxima of AC1 are highlighted by the triangles as in the experimental results in Figure 5.

The results for both rescalings exhibited a modulation similar to what was shown in Figure 9: however, the maxima and minima were no longer at *SM*_par_ and *SM*_ort_. Comparing these results with the experimental data in Figure 5 shows that the skewing in the scaling where α = 2 was very similar to the data. The maximum is to the left of *SM*_par_ and the minimum is close to *SM*_ort_. This skewing was relatively unaffected by the concomitant scaling of the solution manifold. Additional simulations were run where we rescaled all data after the simulations were completed showed similarly skewed modulations. These modeling results suggest that the experimental deviations from the hypothesized pattern can be ascribed to such scaling in the variables at one stage of the processing. However, as the model is a simplification of the actual system dynamics, we do not venture to equate this model exercise with the actual variable scaling in the central nervous system.

## Discussion

The hypothesis that humans are sensitive to the direction of the solution manifold has found support in several lines of research that examined variability with respect to task-relevant and irrelevant dimensions. Using the skill of goal-directed throwing, our experimental and modeling work presents new results that reveal how practice changes both the distributional and temporal structure of data. Further, our new analysis method highlights an important issue: variability analysis is sensitive to the coordinates. As we do not know the coordinates that the CNS operates in, results may be skewed.

We summarize our results with respect to the four hypotheses: (1) Tolerance and Covariation increased with practice, and T-Cost and C-Cost correlated with the decreasing error; Noise, estimated as N-Cost, remained constant. (2) The temporal dynamics of the trial-by-trial data exhibited preferred directions; the structure showed mostly persistence, as quantified by positive autocorrelations and a scaling index greater than 0.5. (3) Six days of practice not only led to improvement in overt performance, but also to an increasing directionality in the temporal structure in execution variable. Model results suggest that this change can be ascribed to increases in the feedback gain. (4) The directions with maximum and minimum structure in the fluctuations were not coincident with the directions orthogonal and parallel to the solution manifold. Simulations of a simplified model of the skittles task helped to interpret these findings. Similar deviation were obtained when applying a simple linear rescaling to one of the state variables.

### Tolerance, covariation, and noise

The decomposition of variability into Tolerance, Covariation, and Noise revealed that the main contribution to practice-induced decrease of error stemmed from Tolerance and Covariation, as estimated by T-Cost and C-Cost. Noise or N-Cost remained constant throughout the 6 days. These differential results for the three components highlighted that practice-induced decreases in variability, commonly quantified by decreasing standard deviations of error or other performance variables, should not be immediately equated with a reduction of stochastic processes. While the present data suggested that stochastic processes were not affected by practice, previous results on 15 practice sessions gave evidence that reduction in noise processes may just have a very slow time scale (Cohen and Sternad, 2009). As in previous studies, Tolerance was a significant factor contributing to error reduction and dropped early in practice. T-Cost quantifies how the data mean, or location in execution space changed with practice. To account for this change in the mean, the directionality analysis was centered for each individual and each block. Covariation had a slower time scale but also significantly contributed to performance improvement. The different time scales of the three components probably reflect the multiple time scales of plastic changes in the nervous system (Kiebel et al., 2008). Note that this parsing of variability into Tolerance, Covariation, and Noise is unique to the TNC-approach. Analyses that focus on the anisotropy using covariance-based methods with respect to mean performance cannot parse the overall decrease in noise, nor detect a possible bias (Latash et al., 2002; Latash, 2008). The fact that Covariation became more pronounced provided the basis for the analysis of temporal fluctuations in different directions.

### Directionality and persistence in temporal dynamics

The trial-to-trial dynamics in the directional execution variables showed a clear modulation of structure in different directions, supporting the overall hypothesis that humans are sensitive to the orientation of the solution manifold. This result is consistent with Dingwell's and van Beers' results, although the studies differ in the kind of structure seen in orthogonal and parallel directions. Initially, negative correlations were expected orthogonal to the solution manifold, compared to persistence in the goal-irrelevant direction, as was reported by Dingwell and colleagues in their study on treadmill walking (Dingwell et al., 2010). In contrast, our study revealed positive autocorrelations in both orthogonal and parallel directions, similar to what van Beers et al. (2013) report for three different tasks. One possible reason for Dingwell's results could be that successive strides are not independent, and the temporal sequence of strides can induce negative autocorrelations. For example, any small measurement error in temporally adjacent variables, such as overestimating one stride length, has the inverse effect on the next stride and underestimates the next stride. Similarly, inertial “carry-over” effects can also enhance this observation. See also the “clock-motor” model on rhythmic timing by Wing and Kristofferson where the effect of noise creates negative lag-1 autocorrelations into the sequence of inter-response intervals. As the authors point out, these negative autocorrelations are simply due to the temporal adjacency of intervals in the presence of a noisy “clock,” not corrective feedback processes (Wing and Kristofferson, 1973a,b).

As the model simulations made explicit, the effect of two added noise sources could lead to positive autocorrelation obscuring the effect of possible corrections. Negative autocorrelations only emerged when the feedback gain became relatively large. One other potential account for the persistence in the data is that subjects did not have direct error information. One challenge in the skittles task is that the visible error is non-linearly mapped onto the execution variables position and velocity at ball release. Hence, subjects may try a “blind” gradient descent to find the best release parameters. Previous studies suggested that when knowledge of results was withheld or when visual information was occluded, the temporal structure of the task output was not white noise but had persistent characteristics (Blackwell and Newell, 1996; Baddeley et al., 2003; Miyazaki et al., 2004). The fact that in our study the scaling index and the autocorrelations showed a consistent pattern gives evidence that there were both short-range and long-range correlations, the latter reflecting system-inherent “memory processes” (Hausdorff et al., 1995). However, without further modeling, the exact nature of these processes remains elusive.

### Practice-induced changes in temporal dynamics

Our study is the first to show that the directional structure in trial-to-trial dynamics changed with practice. The recent study by Dingwell et al. (2012) on learning a virtual reaching task with two different solution manifolds, defined by the product and ratio of reach time and distance, reported a learning effect across 2 days only in the overt error and variance, not in the directionality of temporal structure. This may be due to the fact that the GEM analysis was only performed across 2 days, excluding the initial practice period. Using the rotation analysis, our study showed that the directional modulation in both autocorrelation and scaling index became more pronounced with practice. The initial lack of modulation reflects that subjects did not yet know the directionality of execution space. This is not surprising, as in the skittles task the solution manifold is not visible to the performer but is defined by the mediating dynamics of the ball trajectory. Without knowledge of the orientation of the solution manifold, exploration is needed that may occur in a gradient-descent-like fashion that leads to the persistent structure, as mentioned above. After this exploratory stage, trial-to-trial dynamics became more directionally sensitive and the structure in the orthogonal direction changed from initially positive autocorrelations to white noise and eventually very small negative values.

This result could be replicated with the simple model by a suitably chosen noise ratio and feedback gain. Given that the noise component in the experimental data was constant throughout the 6 days, the noise ratio was fixed to 0.08; assuming further an initially small or zero feedback gain, an increase in the gain to ~0.20 reproduced the experimental modulation of temporal dynamics. Both the decrease in AC1 in the orthogonal direction and the relative invariance in the parallel direction could be replicated in the model results.

### Directionality of temporal structure and sensitivity to coordinates

One important caveat for many approaches that analyze structure of variability is that these analyses are fundamentally sensitive to the chosen coordinates (Müller et al., 2007; Smeets and Louw, 2007; Sternad et al., 2010; Campolo et al., 2013). As demonstrated in our earlier study, variability analyses that rely on the covariance matrix are highly sensitive to the definition of the variables that span the space. This caveat holds for the large array of well-established methods, ranging from principal component analysis to isomap and others. While the mathematical tools are not questioned, when applying these methods to analyze hidden variables used by the nervous system, potential pitfalls arise. How easily the results can be thwarted was highlighted at the example of a UCM analysis of a multi-joint pointing task (Sternad et al., 2010). This study illustrated that results from two different, but equally valid mathematical definitions of joint angles—which are related by a simple linear transformation—differed: a synergy was indicated by anisotropy in one joint space, while not in the other. As shown in our study, the TNC-analysis is also not immune to this problem, but the sensitivity of the three components is less severe, due to the fact that structure of variability is evaluated in result space defined by the task (Campolo et al., 2013).

A second limitation of a covariance-based decomposition of variance in execution space is that they can only be applied in a space that has a defined metric, and thereby, orthogonality. The execution space in the skittles task is defined by angle and velocity, which have different units and, hence, no metric. This is similar to the GEM-analysis of walking, where the space was spanned by stride length and period. A straightforward remedy is to normalize the variables by their variance, as was done by Dingwell and colleagues and also in our skittles analysis. However, this correction by no means guarantees the right metric from which to define orthogonality. The fundamental issue is that the analyses rely on the assumption that the chosen execution variables span the space that is relevant for the nervous system. Until we know the coordinates used by the nervous system, this remains a tenuous assumption (Lacquaniti and Maioli, 1994; Fasse et al., 2000).

Clearly, there is no easy remedy. For the analysis of our experimental data we first normalized the coordinates by their variance before conducting time series analyses. We then introduced a rotation analysis that did not a priori depend on the definition of orthogonality but scouted the data for the direction with structure that may be relevant for the controller. The results showed that the direction of maximum persistence was not exactly parallel to the solution manifold and also did not significantly differ from the angle direction. The direction of minimum structure was coincident with the orthogonal direction and did differ from velocity, although only after some practice. These deviations from the straightforward expectations may be accounted for by the fact that the variables measured in external coordinates do not have to map onto the variables used by the nervous system.

To demonstrate such possible distortions, we used the simple model and introduced a linear rescaling of one of the state variables to skew the directions of maximum and minimum temporal correlations. Importantly, such rescaling can take place at many stages of the system: It can be applied at each iteration loop inside the system, it can happen independent of a concomitant rescaling of the solution manifold, it can include or exclude the noise, or it may only be applied on the data distributions. We modeled some select possibilities. The results showed that a linear rescaling of one variable indeed produced a skewing of the directionality of the data. Interestingly, this rescaling closely replicated the observed distortions in the pattern of modulation in the experimental data. While we did not intend to quantitatively model the experimental data, the results illustrate that the observed deviations in the directionality of the temporal structure may be caused by such internal rescaling. We venture the speculation that such results may provide clues about the relative scaling of the coordinates inside the nervous system.

## Conclusions

In summary, this experimental and modeling work demonstrated that the acquisition of a complex motor skill with a redundant task space is associated with an increasing anisotropy in data distributions and a corresponding increase in directionality in their temporal structure. The experimental results showed that the Tolerance, Covariation, and Noise of the data distributions changed with different time scales. The time-dependent characteristics in execution variables give further support that trial-to-trial dynamics is structured in directions specific to the solution manifold. A new analysis method highlighted that an a priori assumption of orthogonality in execution space may thwart the results. Model simulations suggested that the performance improvement is largely accounted for by changes in one essential system parameter—feedback gain. Further, analysis of the model highlighted how a rescaling of the variables can thwart the directionality of the maximum temporal correlations. These results may encourage future studies on variability to be less reliant on predefined directions. Rather, the search for directionality could help to reveal the coordinates important to the central nervous system.

### Conflict of interest statement

The authors declare that the research was conducted in the absence of any commercial or financial relationships that could be construed as a potential conflict of interest.

## References

[B1] Baddeley R. J. Ingram H. A. Miall R. C. (2003). System identification applied to a visuomotor task: near-optimal human performance in a noisy changing task. J. Neurosci. 23, 3066–3075 12684493 10.1523/JNEUROSCI.23-07-03066.2003 PMC6742112

[B2] Blackwell J. R. Newell K. M. (1996). The informational role of knowledge of results in motor learning. Acta Psychol. 92, 119–129 10.1016/0001-6918(95)00013-5 8712035

[B4] Campolo D. Widjaja F. Xu H. Ang W. T. Burdet E. (2013). Analysis of accuracy in pointing with redundant hand-held tools: a geometric approach to the uncontrolled manifold method. PLoS Comput. Biol. 9:e1002978 10.1371/journal.pcbi.1002978 23592956 PMC3617015

[B5] Cohen R. G. Sternad D. (2009). Variability in motor learning: relocating, channeling and reducing noise. Exp. Brain Res. 193, 69–83 10.1007/s00221-008-1596-1 18953531 PMC2756422

[B6] Cohen R. G. Sternad D. (2012). State space analysis of intrinsic timing: exploiting task redundancy to reduce sensitivity to timing. J. Neurophysiol. 107, 618–627 10.1152/jn.00568.2011 22031769 PMC3349626

[B7] Cusumano J. P. Cesari P. (2006). Body-goal variability mapping in an aiming task. Biol. Cybern. 94, 367–379 10.1007/s00422-006-0052-1 16501988

[B8] Dingwell J. B. John J. Cusumano J. P. (2010). Do humans optimally exploit redundancy to control step variability in walking? PLoS Comput. Biol. 6:e1000856 10.1371/journal.pcbi.1000856 20657664 PMC2904769

[B9] Dingwell J. B. Smallwood R. F. Cusumano J. P. (2012). Trial-to-trial dynamics and learning in a generalized redundant reaching task. J. Neurophysiol. 109, 225–237 10.1152/jn.00951.2011 23054607 PMC3545167

[B10] Eke A. Herman P. Kocsis L. Kozak L. R. (2002). Fractal characterization of complexity in temporal physiological signals. Physiol. Meas. 23, R1–R38 10.1088/0967-3334/23/1/201 11876246

[B11] Faisal A. A. Selen L. P. Wolpert D. M. (2008). Noise in the nervous system. Nat. Rev. Neurosci. 9, 292–303 10.1038/nrn2258 18319728 PMC2631351

[B12] Fasse E. D. Hogan N. Kay B. A. Mussa-Ivaldi F. A. (2000). Haptic interaction with virtual objects. Biol. Cybern. 82, 69–83 10650909 10.1007/PL00007962

[B13] Feder J. (1988). Fractals. New York, NY: Plenum Press

[B14] Hausdorff J. M. Peng C. K. Ladin Z. Wei J. Y. Goldberger A. L. (1995). Is walking a random walk? Evidence for long-range correlations in stride interval of human gait. J. Appl. Physiol. 78, 349–358 7713836 10.1152/jappl.1995.78.1.349

[B15] Kiebel S. J. Daunizeau J. Friston K. J. (2008). A hierarchy of time scales in the brain. PLoS Comput. Biol. 4:e1000209 10.1371/journal.pcbi.1000209 19008936 PMC2568860

[B16] Lacquaniti F. Maioli C. (1994). Coordinate transformations in the control of cat posture. J. Neurophysiol. 72, 1496–1515 7823082 10.1152/jn.1994.72.4.1496

[B17] Latash M. L. (2008). Synergy. Oxford, UK: Oxford University Press

[B18] Latash M. L. (2010). Stages in learning motor synergies: a view based on the equilibrium-point hypothesis. Hum. Mov. Sci. 29, 642–654 10.1016/j.humov.2009.11.002 20060610 PMC2891849

[B19] Latash M. L. Scholz J. P. Schöner G. (2002). Motor control strategies revealed in the structure of motor variability. Exerc. Sport Sci. Rev. 30, 26–31 11800496 10.1097/00003677-200201000-00006

[B20] Miyazaki M. Nakajima Y. Kadota H. Chitose K. Ohtsuki T. Kudo K. (2004). 1/f-type fluctuation in human visuomotor transformation. Neuroreport 15, 1133–1136 15129160 10.1097/00001756-200405190-00010

[B21] Müller H. Frank T. Sternad D. (2007). Variability, covariation and invariance with respect to coordinate systems in motor control. J. Exp. Psychol. Hum. Percept. Perform. 33, 250–255 10.1037/0096-1523.33.1.250 17311492

[B22] Müller H. Sternad D. (2004). Decomposition of variability in the execution of goal-oriented tasks—Three components of skill improvement. J. Exp. Psychol. Hum. Percept. Perform. 30, 212–233 10.1037/0096-1523.30.1.212 14769078

[B23] Müller H. Sternad D. (2009). Motor learning: changes in the structure of variability in a redundant task, in Progress in Motor Control—A Multidisciplinary Perspective, ed Sternad D. (New York, NY: Springer), 439–456 10.1007/978-0-387-77064-2_23 PMC3776417 19227514

[B24] Oldfield R. C. (1971). The assessment of handedness: the Edinburgh inventory. Neuropsychologica 9, 97–113 5146491 10.1016/0028-3932(71)90067-4

[B26] Scholz J. P. Schöner G. (1999). The uncontrolled manifold concept: identifying control variables for a functional task. Exp. Brain Res. 126, 289–306 10382616 10.1007/s002210050738

[B27] Smeets J. B. J. Louw S. (2007). The contribution of covariation to skill improvement is an ambiguous measure. J. Exp. Psychol. Hum. Percept. Perform. 33, 246–249 10.1037/0096-1523.33.1.246 17311491

[B28] Sternad D. Abe M. O. Hu X. Müller H. (2011). Neuromotor noise, sensitivity to error and signal-dependent noise in trial-to-trial learning. PLoS Comput. Biol. 7:e1002159 10.1371/journal.pcbi.1002159 21966262 PMC3178634

[B29] Sternad D. Park S. Müller H. Hogan N. (2010). Coordinate dependency of variability analysis. PLoS Comput. Biol. 6:e1000751 10.1371/journal.pcbi.1000751 20421930 PMC2858681

[B30] Stimpel E. (1933). Der Wurf (On throwing), in Motorik (On motor control), eds Krüger F. Klemm O. (München, Germany: Beck), 109–138

[B31] Todorov E. (2004). Optimality principles in sensorimotor control. Nat. Neurosci. 7, 907–915 10.1038/nn1309 15332089 PMC1488877

[B32] Todorov E. Jordan M. I. (2002). Optimal feedback control as a theory of motor coordination. Nat. Neurosci. 5, 1226–1235 10.1038/nn963 12404008

[B33a] van Beers R. J. (2009). Motor learning is optimally tuned to the properties of motor noise. Neuron 63, 406–417 10.1016/j.neuron.2009.06.025 19679079

[B33] van Beers R. J. Brenner E. Smeets J. B. J. (2013). Random walk of motor planning in task-irrelevant dimensions. J. Neurophysiol. 109, 969–977 10.1152/jn.00706.2012 23175799

[B34] Verrel J. Pradon D. Vuillerme N. (2012). Persistence of motor-equivalent postural fluctuations during bipedal quiet standing. PLoS ONE 7:e48312 10.1371/journal.pone.0048312 23110228 PMC3482199

[B35] Wing A. M. Kristofferson A. B. (1973a). Response delays and the timing of discrete motor responses. Percept. Psychophys. 14, 5–12 10.3758/BF03198607

[B36] Wing A. M. Kristofferson A. B. (1973b). The timing of interresponse intervals. Percept. Psychophys. 1, 455–460 10.3758/BF03205802

